# Effect of a symbiotic microbial complex (SMC) on productivity, early viability and blood parameters in broiler chicken under stress rearing conditions

**DOI:** 10.5455/javar.2026.m1021

**Published:** 2026-03-13

**Authors:** Lorena Del Carmen Vivas Ríos, Neyo De La Cruz Pérez Guedez, Marlon Francisco Brazon Lunar, Moraiza Josefina Casado Chacín, Anthony Deison Mendoza Sandoval, David Santiago Coll, Hennet José Faria Villarreal, Danilo Jesús Zavala López, Alberto José Quintero

**Affiliations:** 1 Animal Nutrition and Feeding Laboratory, Centre for Agricultural Biotechnology, Venezuelan Institute for Scientific Research, Carretera Panamericana, Km 11, Miranda State, Bolivarian Republic of Venezuela; 2 Computational Chemistry Laboratory, Chemistry Centre, Venezuelan Institute for Scientific Research, Carretera Panamericana, Km 11, Miranda State, Bolivarian Republic of Venezuela; 3 Liofilizados de Venezuela, C.A., Av. Circunvalación 2, Maracaibo, State Zulia, Bolivarian Republic of Venezuela; 4 Animal Genetics and Reproduction Laboratory, Centre for Agricultural Biotechnology, Venezuelan Institute for Scientific Research, Carretera Panamericana, Km 11, Miranda State, Bolivarian Republic of Venezuela

**Keywords:** Animal performance, *Bacillus*, homeostasis, non-nutritive additive, metabolic resilience, serum biochemistry

## Abstract

**Objectives:** This study evaluated the effects of a novel Symbiotic Microbial Complex (SMC) as a non-nutritive additive on the productive performance, hematological parameters, and serum biochemistry of broiler chickens reared under environmental stress.

**Materials and Methods:** A total of 264 one-day-old Ross broiler chickens were assigned to two treatments: A Control and an SMC treatment (10% dietary inclusion), with three replicates of 44 birds each, for 42 days. Diets were isoproteic and isoenergetic for the Pre-starter (1–10 days), Starter (11–22 days), and Finisher (23–42 days) phases. Environmental parameters (averaging 29.6°C) and water quality (Nitrates) were monitored. Weekly evaluations of production variables, hematology, and serum biochemistry were performed. Data were analyzed using one-way ANOVA and Chi-square tests.

**Results:** Inclusion of SMC significantly reduced cumulative mortality in the Pre-starter (20.45% Control vs. 3.03% SMC) and Starter phases (6.06% Control vs. 0.00% SMC) (*p* < 0.05). While no significant differences were observed in body weight or feed conversion ratio, the SMC treatment exhibited greater physiological stability. Liver enzyme activity (ALT/AST) was lower in SMC birds during metabolic peaks, and lipid profiles remained within normal physiological ranges despite environmental challenges.

**Conclusions:** The SMC acted as a potent bioprotector, significantly enhancing early viability and maintaining systemic homeostasis. Its protective effect buffered the physiological toll of environmental heat and suboptimal water quality. However, its growth-promoting potential may be optimized under controlled environmental settings where metabolic energy is not prioritized for survival and homeostatic maintenance.

## 1. Introduction

The poultry industry is constantly seeking sustainable methods to increase broiler production. Traditionally, antibiotic growth promoters have been key, but concerns about drug resistance have prompted the search for alternatives [1]. Additives such as probiotics have emerged as a promising strategy to promote growth, health, and productivity in broiler chickens [2]. Defined as live microorganisms that benefit the host, probiotics can improve the productive performance of broilers, including body weight, weight gain, feed conversion ratio, and mortality [3, 4, 5, 6, 7, 8, 9, 10, 11]. Furthermore, they facilitate digestion, optimize the intestinal microbiome, and strengthen bird immunity [8]. Understanding their impact is essential to developing feeding strategies that promote more efficient, healthier poultry production.

Symbiotic, defined as a synergistic combination of probiotics and prebiotics, improves the survival rate and microbial balance in the gastrointestinal tract of broiler chickens, often exceeding the benefits of administering them separately. In this study, prebiotics are considered essential non-nutritive additives that promote the microbiota in the gastrointestinal tract [12]. β-glucans stand out as a vital component of all these prebiotic sources; they activate macrophages, stimulate the reticuloendothelial system, and significantly enhance antibody production, thereby increasing host resistance to infection. Additionally, β-mannanase has shown potential as a cost-saving measure by optimizing nutrient absorption and energy utilization in soybean-based diets [13, 14].

Despite these benefits, poultry production in tropical regions faces severe challenges due to environmental stressors, such as high ambient temperatures and suboptimal water quality, specifically high nitrate levels, which induce physiological stress and compromise bird health. There is a lack of research evaluating how specific, novel formulations perform under these real-world field stress conditions.

The purpose of this study was to examine the effect of a novel Symbiotic Microbial Complex (SMC), a specific non-nutritive additive formulated by LIVENCA consisting of five Bacillus strains and a prebiotic blend, on the productive performance and physiological resilience of Ross broilers. This research specifically evaluated the SMC’s influence on zootechnical factors and blood parameters under current tropical rearing conditions, including water quality and environmental heat.

## 2. Materials and Methods

### 2.1. Ethical approval

In compliance with ethical standards and institutional and national guidelines for the care and use of laboratory animals, the Bioethics Committee for Animal Research of the Venezuelan Institute for Scientific Research (COBIANIM 2025-02; May 13, 2025) was followed.

### 2.2. Experimental design, animals, and diets

The 42-day study was conducted at the Nicolino Ierrobueno Agricultural Production Unit in Maracay, Aragua state, Venezuela. A total of 264 one-day-old Ross broilers (mixed sex) were obtained from a local commercial hatchery and randomly assigned to two treatments (132 broilers each), with three replicates of 44 birds per treatment. The birds were fed a three-phase feeding program: Pre-starter (1–10 days), Starter (11–22 days), and Finisher (23–42 days).

Diets were formulated to be isoproteic and isoenergetic. To ensure optimal growth potential, crude protein levels were strictly adjusted to Ross nutritional specifications [15]. with 23.0% for Pre-starter, 21.5% for Starter, and 19.5% for Finisher phases. Metabolizable energy values were calculated based on the nutritional matrices of the ingredients according to NRC [16] standards, aiming for targets of 2975, 3050, and 3100 kcal/kg, respectively. The analyzed proximate composition of the experimental diets, performed according to the Official Methods of Analysis of the Association of Official Analytical Chemists [17], is presented in Table 1. Minor variations between treatments are attributed to inherent variability in the raw material.

**Table 1.** Determination of the proximal composition of experimental diets.

Parameters	Pre-Starter		Starter		Finisher	
Calculated levels (Formulation)						
*Protein (%)	23.0		21.5		19.5	
**Metabolizable energy (kcal/kg)	2975		3050		3100	
**Analyzed composition (Laboratory)**	**Control**	**SMC**	**Control**	**SMC**	**Control**	**SMC**
Moisture (%)	9.50	9.62	9.35	9.47	11.42	11.05
Protein (%)	19.36	20.01	19.38	20.12	19.44	19.38
Fat (%)	7.88	6.69	10.27	9.85	9.56	9.66
Ash (%)	9.36	7.14	6.15	5.58	5.46	5.55
Fiber (%)	2.42	2.73	2.27	2.37	2.68	2.55
Calcium (%)	1.60	1.42	1.19	1.08	0.90	1.01
Phosphorus (%)	0.68	0.67	0.60	0.63	0.62	0.57

* Crude protein levels were formulated to meet Ross specifications [15]. ** Metabolizable energy levels were estimated by calculations based on the nutrient composition of the ingredients according to NRC [16] standards.

The Control treatment received a standard commercial diet (soybean meal, offal meal, L-methionine, L-threonine, soybean oil, choline chloride, vitamin-minerals premix). The experimental treatment consisted of the same basal formulation with the dietary incorporation of 10% Symbiotic Microbial Complex (SMC). The SMC is a novel formulation consisting of five bacterial strains (*Bacillus subtilis* 8–12%, *Bacillus toyonensis* 8–12%, *Bacillus licheniformis* 13–17%, *Heyndrickxia coagulans* 8–12%, and *Shouchella clausii* 18–22%) and prebiotics (β-glucans 3–7% and β-mannanase 3–7%). These strains were molecularly identified via conventional PCR at the University of Zulia (Maracaibo, Venezuela), and SMC was provided by the LIVENCA laboratory (Maracaibo, Venezuela).

### 2.3. Rearing conditions and environmental stress

Chickens were housed in experimental pens (4.84 m^2^) with rice hull as bedding material at a density of 10 chickens/m^2^. Each pen was equipped with infrared heat lamps, manual bell-type waterers, and tube feeders, which were provided throughout the study. *Ad libitum* access to water and feed was provided. Environmental parameters were monitored using a digital thermohygrometer (Extech Instruments, NH, USA, model 445715); the average ambient temperature was 29.6°C, and the relative humidity was 57.3%, indicating high–temperature tropical rearing stress.

To evaluate the SMC effect under real field stress, drinking water was not treated. Physicochemical analysis [18] revealed suboptimal quality: absence of residual free chlorine (< 0.3 mg/l), slightly acidic pH (6.32), and high nitrate levels (34.53 mg/l NO_3_-), which exceed the recommended safety limit for poultry.

### 2.4. Productive variables

Production variables were monitored throughout the 42-day experimental period, including initial body weight (IBW), final body weight (FBW), daily feed intake (DFI), body weight gain (BWG), feed conversion ratio (FCR), and mortality percentage. A total of 15 chickens per treatment were randomly selected and individually identified for weighing on days 1, 4, 7, 10, 14, 21, 22, 28, 35, and 42. Although body weight was recorded frequently to comply with the Ross management manual guidelines [15] (Days 4 and 10), and to monitor physiological status during weekly blood sampling, the productive parameters presented in Table 2 were calculated using only the weights recorded at the beginning and the end of each dietary phase (Day 1, 10, 22, and 42). All measurements were performed using a precision scale (CGoldenwall, Hangzhou, China, model HZ50002B, 0.01 gm sensitivity).

**Table 2.** Effects of Symbiotic Complex (SMC) and rearing phase on broiler production performance and cumulative mortality.

Parameter/Treatment	Pre-starter		Starter		Finisher	
**Control**	**SMC**	**Control**	**SMC**	**Control**	**SMC**
IBW (gm)	38.64 ± 0.83	39.26 ± 0.76	265.80 ± 81.57	271.07 ± 84.30	1092.07 ± 321.45	1087.20 ± 306.18
FBW (gm)	265.80 ± 81.57	271.07 ± 84.30	1092.07 ± 321.45	1087.20 ± 306.18	2743.88 ± 347.58	2649.51 ± 415.99
DFI (gm)	25.11 ± 7.59	40.19 ± 10.05	98.95 ± 20.33	84.27 ± 27.5	214.90 ± 22.77	194.71 ± 10.17
BWG (gm)	227.16	231.80	826.27	816.13	1651.81	1562.31
FCR	0.75	0.36	0.99	0.80	1.53	1.91
Mortality (%)	20.45^b^	3.03^a^	6.06^b^	0.00^a^	0.00	1.52

Data are presented as Mean ± Standard Deviation (SD). Different superscripts (a, b) within the same row and phase indicate significant differences *p* < 0.05. IBW, initial body weight; FBW, final body weight; DFI, daily feed intake; BWG, body weight gain; FCR, feed conversion ratio.

The variables were calculated using the following formulas:


1 \[
{\mathrm{DFI = }}\frac{{{\mathrm{Feed\ offered\ (gm) - Feed\ not\ consumed(gm)}}}}{{{\mathrm{Total\ number\ of\ animals}}}}
\]

DFI: Daily feed intake.


2 \[
{\mathrm{IWG = FBW}}\left({{\rm gm}} \right){\rm - IBW(gm)}
\]

BWG: Body weight gain; IBW: Initial body weight; FBW: Final body weight.


3 \[
{\mathrm{FCR = }}\frac{{{\mathrm{Quantity\ of\ feed\ consumed\ during\ a\ period\ (gm)}}}}{{{\mathrm{Weight\ gain\ during\ the\ same\ period\ (gm)}}}}
\]

FCR: Feed conversion ratio.


4 \[
{\mathrm{\% Mortality = }}\frac{{{\mathrm{Total\ deaths \times 100}}}}{{{\mathrm{Total\ population}}}}
\]

### 2.5. Sampling and analysis of blood parameters

Blood samples were collected weekly via cardiac puncture. Due to logistical limitations and the exploratory nature of monitoring, biochemical and hematological parameters were evaluated descriptively by sampling one representative bird per treatment each week (*n* = 1); consequently, these results are presented as a time series to monitor physiological trends rather than through comparative statistics. Biochemistry was analyzed weekly starting from day 7 and included the following parameters: Blood glucose, urea, creatinine, alanine aminotransferase (ALT), aspartate aminotransferase (AST), total bilirubin, total protein, blood calcium, cholesterol, and triglycerides. Complete blood counts were performed weekly from day 14, including total leukocytes, segmented neutrophils (heterophils), lymphocytes, eosinophils, and monocytes. All analytical procedures followed ISO 15189 [19] standards for the quality of clinical laboratories.

### 2.6. Statistical analysis

Production variables were analyzed using Statistical Product and Service Solutions (SPSS) IBM version 21 [20]. A one-way Analysis of Variance (ANOVA) was performed, with treatment and phase as fixed factors, according to the following model:


5 \[
{{\mathrm{Y}}_{\mathrm{i}}} = {\mathrm{\mu }} + {{\mathrm{T}}_{\mathrm{i}}} + {{\mathrm{\varepsilon }}_{\mathrm{i}}}
\]

Where: Y_i_ is the observed response in the ith unit, μ is the overall mean, T_i_ is the effect of the i^th^ treatment, ε_i_ is the random error term. Mortality data were analyzed using the *Chi*-square (χ^2^) test. Hematological and biochemical data (Tables 3, 4) were evaluated using the percentage difference (Δ%) between the SMC and Control. This descriptive time–series approach was implemented to characterize the magnitude and trend of the physiological response under stressful rearing conditions.

## 3. Results and Discussion

### 3.1. Productive performance and early viability

**Figure 1.**
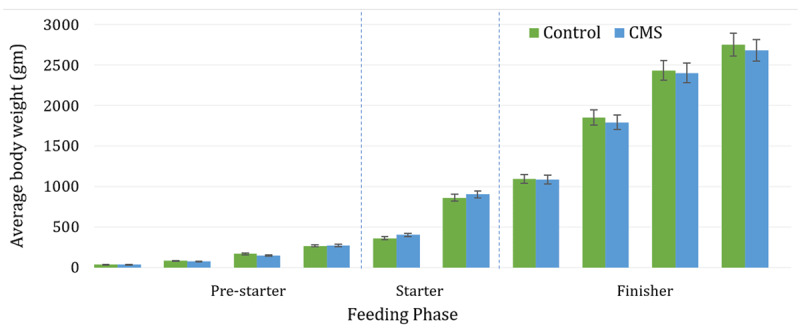
Evolution of the average body weight of broilers.

The effects of SMC administration on the productive performance of Ross broilers are summarized in Table 2. No significant differences (*p* > 0.05) were found between the Control and the SMC treatments for IBW, FBW, DFI, and BWG across the different phases. Despite the lack of significant variance in growth parameters, the evolution of body weight demonstrated that both treatments maintained a steady growth curve consistent with the breed′s standards, even under the environmental stress conditions described (Figure 1).

In contrast to the growth variables, the mortality rate showed significant differences (*p* < 0.05). The administration of the SMC resulted in a significant reduction in mortality during the Pre-starter and Starter phases compared to the Control. These results indicate that while the SMC did not alter the final weight of the birds, it significantly enhanced early viability, allowing a higher percentage of the flock to reach the end of the production cycle under challenging rearing conditions.

The lack of significant differences (*p* > 0.05) in final body weight and feed intake suggests that under the specific conditions of this study, the symbiotic complex acted as a physiological stabilizer rather than a growth promoter. This is consistent with findings by Ciurescu et al. [21] and Niu et al. [22], who observed that the supplementation of *Bacillus subtilis* and *Bacillus coagulans* does not always result in superior body weight when birds are raised under slightly challenging conditions, as the probiotic effect often prioritizes metabolic efficiency and survival over mass accumulation.

Furthermore, the growth kinetics confirm that the birds reached their genetic potential, as defined by Ross standards. Even under environmental stressors such as heat and nitrate-contaminated water, the SMC maintained a steady growth trajectory. This resilience effect is supported by Khongthong et al. [23], who demonstrated that *Bacillus subtilis* maintains gut barrier integrity and performance in stressful tropical environments by modulating the inflammatory response.

The reduction in mortality (*p* < 0.05) during the Pre-starter and Starter phases aligns with recent findings by Rodrigues et al. [24] and Ningsih et al. [25], who demonstrated that *Bacillus*-based probiotics mitigate the adverse effects of enteric challenges and environmental stress. Furthermore, Pewan et al. [26] emphasize that these microbial strategies are essential alternatives to antibiotics, significantly enhancing early viability and immune robustness in poultry facing multifactorial stressors.

In this study, the challenging environment was characterized by suboptimal water quality (high nitrate levels and no chlorine) and high ambient temperatures (29.6°C). Under these rearing stress conditions, the SMC likely acted as a bioprotective barrier. This is consistent with Khongthong et al. [23], who reported that probiotic strategies are vital for mitigating heat stress effects on broiler performance by maintaining gut barrier integrity and reducing the inflammatory response in stressful tropical environments.

The presence of nitrates in the drinking water is a known stressor that induces oxidative stress and alters the cecal microbiota [27, 28]. According to Li et al. [29] and Mohamed et al. [30], water quality is the primary driver of early gut colonization. In the Control treatment, the cumulative effect of nitrates and tropical heat likely led to dysbiosis and a higher rate of opportunistic infections. This environmental pressure can trigger a persistent proinflammatory response, as described by White et al. [31], in which environmental stressors activate molecular pathways (such as NF-kB) that compromise systemic homeostasis.

In contrast, the SCM treatment benefited from the competitive exclusion and immune modulation provided by *Bacillus licheniformis, Bacillus subtilis*, and *Bacillus clausii*. These strains, combined with the prebiotic action of mannans and β-glucans, enhance the intestinal barrier and reduce the impact of pathogens even under severe heat stress [23, 32, 33]. As noted by Polidoro et al. [34] and Sanmiguel et al. [35], mannan-oligosaccharides (MOS) serve as an effective alternative to antibiotic growth promoters by preventing the attachment of pathogenic bacteria to the intestinal mucosa during the critical first 14 days of life [36, 37]. This intestinal protection is further enhanced by the inclusion of *Bacillus strains*, which promote a healthier gut environment and improve the zootechnical response during early development [38].

Ultimately, while the SMC did not produce heavier birds, it ensured a higher survival rate. This improvement in early viability underscores the complex’s role as a critical management tool for mitigating economic losses in poultry production systems facing water-quality challenges and tropical heat stress [10, 39]. Current research supports the view that these microbial additives serve as viable alternatives to traditional antibiotics, especially in regions with prevalent environmental stressors, by stabilizing hematobiochemical dynamics and ensuring a more resilient flock [3, 26].

### 3.2. Hematological parameters

Regarding the hematological parameters, although a descriptive analysis was performed (*n* = 1), the observations in the SMC treatment suggest a more robust immune response (Table 3). The faster recovery of total leukocyte counts in supplemented birds after the initial challenge (Days 14 and 21) coincides with the findings of Bogatko [41], who observed an increase of 4.4% to 17.2% in leukocyte counts in broilers treated with probiotics. Furthermore, maintaining blood parameters within the physiological ranges established for modern Ross broilers [42] confirms that the SMC supports homeostatic stability even under stress.

**Table 3.** Weekly descriptive monitoring of hematological parameters in broiler chickens supplemented with a symbiotic microbial complex (SMC).

Parameter	Day	Control	SMC	Difference (Δ%)	Reference values*
Leucocytes (cell/µl)	14	90871	75918	–16,5	4000–40000
	21	59583	47376	–20,5	
	28	21450	28200	31,5	
	35	31949	51052	59,8	
	42	45117	33796	–25,1	
Segmented (%)	14	35	56	60,0	20–50
	21	46	49	6,5	
	28	50	46	–8,0	
	35	55	46	–16,4	
	42	46	51	10,9	
Lymphocytes (%)	14	62	42	–32,3	45–75
	21	50	46	–8,0	
	28	46	50	8,7	
	35	42	51	21,4	
	42	52	45	–13,5	
Eosinophils (%)	14	1	1	0,0	0–2
	21	2	2	0,0	
	28	2	2	0,0	
	35	1	2	100,0	
	42	1	2	100,0	
Monocytes (%)	14	2	1	–50,0	2–10
	21	2	3	50,0	
	28	2	2	0,0	
	35	2	1	–50,0	
	42	1	2	100,0	

* Reference ranges according to Garfia Veterinary Laboratory [40]. Δ%: Percentage difference of the SMC treatment relative to the Control treatment. Given n = 1 per week, data are presented for descriptive longitudinal monitoring.

Direct comparison between treatments reveals a critical difference in the timing and magnitude of the immune response. While both treatments faced a reduction in leukocytes by Day 28, this reflects a classic response to immune system exhaustion following an acute and prolonged infectious challenge. The SMC treatment maintained a Δ = 31.5% higher count than the Control treatment at this critical point, reflecting greater biological efficiency in the animal.

Furthermore, the SMC birds showed early and robust recovery, reaching a peak by Day 35 (Δ = 59.8% compared to Control), before returning to physiological stability by the end of the study. A faster-recovering immune system can redirect the energy of the immune response toward growth and production (feed efficiency). The SMC treatment mitigates the negative impact of infectious stress on the animal’s overall homeostasis, promoting the restoration of cellular defenses [43, 44, 45]. A high white blood cell count could be related to poor water quality and the possible presence of antinutrients, which induce the production of more antibodies that stimulate the production of white blood cells to fight infection, acting as a defense system [46]. In contrast, the Control treatment showed a delayed increase only by Day 42. This accelerates leucocyte mobilization in the SMC treatment, especially during the finisher transition, is consistent with the immune-modulating effects of probiotics described by Bogatko [41], allowing for a faster resolution of the infectious challenge compared to the delayed response observed in non-supplemented birds.

The importance of lymphocytes lies in their role in adaptive immunity. Table 3 shows that both treatments remain within this range. However, a direct comparison reveals that lymphopenia (a decrease in lymphocytes) was present in the Control treatment on Day 14 (Δ = –32.3%) and 42 (Δ = –13.5%) compared to SMC, which is usually indicative of chronic stress or advanced disease, where cell redistribution or destruction occurs.

Balcerowska and Kwaśnik [47] indicate that chronic stress dysregulates the immune system, impairing its ability to fight infections and accelerating disease progression. Furthermore, Nwaigwe et al. [45] reported that physiological stress depletes the blood cell pool, leading to a reduction in lymphocyte counts. The release of corticosterone during prolonged stress induces a shift from Th1 to Th2 responses, causing lymphocyte apoptosis [47]. In contrast, the lymphocyte dynamics in SMC treatment suggest that adaptive immunity was maintained in a superior state, providing better protection against the rearing challenges. Specifically, the heterophil-to-lymphocyte (H/L) ratio, a recognized biomarker of chronic stress [1], remained more stable in the SMC treatment despite the environmental heat (29.6°C). This stabilization is consistent with Shanmugasundaram et al. [48], who reported that *Bacillus*–based probiotics reduce the hematological impact of heat stress by modulating the homeostatic response. Furthermore, the recovery of lymphocyte populations suggests an enhanced adaptive immune response, likely primed by the β-glucans and mannans in the SMC [23, 33].

Eosinophils for both treatments remained within the reference range (Table 3), consistent with the findings of Nwaigwe et al. [45] and Kareem et al. [49], who evaluated blood parameters as indicators of broiler chicken health.

Regarding segmented neutrophils, which constitute the first line of bacterial defense, a distinct reactive pattern was observed. At the beginning of the study (Day 14), the SMC treatment showed an initial increase (56%), slightly exceeding the reference range (20–50%). However, while the Control treatment eventually surpassed physiological limits during the critical Finisher phase (Day 35, 55%), the SMC treatment demonstrated superior modulation, returning to and remaining within reference levels during that same period (46%).

This suggests that although the SMC treatment responded intensely to the initial environmental challenge, it achieved a more effective homeostatic balance as the cycle progressed. In contrast, the Control treatment showed uncontrolled inflammatory spikes during the period of highest metabolic demand. This capacity of the SMC to regulate the heterophil response and avoid late-stage inflammatory spikes aligns with the findings of Sufiriyanto et al. [43], Kareem et al. [49], and Ávilez et al. [50].

Monocyte values varied within or slightly below the reference range for both treatments (Table 3). A key difference was observed on Day 21: The monocyte peak in the SMC treatment occurred just prior to the highest mortality stress period. This suggests an early and robust activation of the innate immune system in the SMC treatment, allowing for a more rapid resolution of the infectious challenge and explaining the absence of animal losses in this treatment compared to the Control.

Monocytes are precursors of macrophages, cells essential for phagocytosis and antigen presentation. While an increase is a typical response to infection, in the SMC treatment, this early activation (Day 21) correlated directly with survival, effectively modulating the inflammatory response to eliminate pathogens more effectively [43, 45, 49, 50, 51].

### 3.3. Serum biochemistry and metabolic resilience

The serum biochemical profile of Ross broilers is presented in Table 4. Multiple deviations from traditional literature ranges were observed in both treatments, including frequent elevations in urea and total bilirubin, alongside total protein levels often falling within or below the standard reference range. In the SMC treatment, specific peaks in blood glucose, creatinine, and blood calcium were recorded toward the end of the study. As noted by Muthukumaran et al. [12], the inclusion of β-glucans plays a crucial role in maintaining blood glucose homeostasis and stability observed in the SMC treatment.

**Table 4.** Weekly evolution of serum biochemical profiles in broiler chickens (*n* = 1 per treatment/week) under stress conditions.

Parameter	Day	Control	SMC	Difference (Δ%)	Reference values*
Blood glucose (mg/dl)	7	248	253	2.0	200–370
	14	321	232	–27.7	
	21	225	92	–59.1	
	28	195	85	–56.4	
	35	248	102	–58.9	
	42	219	450	105.5	
Urea (mg/dl)	7	5.7	5.2	–8.8	2.50–5.00
	14	1.7	5.5	223.5	
	21	3.9	14.1	261.5	
	28	11.3	14	23.9	
	35	3.6	18.4	411.1	
	42	8.1	12.8	58.0	
Creatinine (mg/dl)	7	0.42	0.26	–38.1	0.17–0.42
	14	0.29	0.33	13.8	
	21	0.38	0.3	–21.1	
	28	0.14	0.23	64.3	
	35	0.18	0.44	144.4	
	42	0.24	0.44	83.3	
ALT (UI/l)	7	10	247	2370.0	20–50
	14	138	22	–84.1	
	21	157	24	–84.7	
	28	150	158	5.3	
	35	260	110	–57.7	
	42	321	358	11.5	
AST (UI/l)	7	8	275	3337.5	25–275
	14	302	147	–51.3	
	21	169	25	–85.2	
	28	165	183	10.9	
	35	270	120	–55.6	
	42	313	348	11.2	
Total bilirubin(mg/dl)	7	0.78	1.27	62.8	0.006–0.12
	14	0.15	1.34	793.3	
	21	0.69	0.25	–63.8	
	28	0.77	0.46	–40.3	
	35	0.46	0.38	–17.4	
	42	0.47	0.24	–48.9	
Total protein (gm/dl)	7	2.59	2.51	–3.1	2.50–4.50
	14	2.06	3.99	93.7	
	21	2.24	3.08	37.5	
	28	2.83	3.01	6.4	
	35	2.43	3.29	35.4	
	42	3.17	3	–5.4	
Blood calcium (mg/dl)	7	9.47	10.9	15.1	8.00–11.00
	14	10.33	12.67	22.7	
	21	10.59	10.12	–4.4	
	28	8.07	10.24	26.9	
	35	11.97	9.39	–21.6	
	42	9.07	12.67	39.7	
Cholesterol (mg/dl)	7	231	184	–20.3	87–192
	14	167	101	–39.5	
	21	142	198	39.4	
	28	174	183	5.2	
	35	141	180	27.7	
	42	89	120	34.8	
Triglycerides (mg/dl)	7	168	201	19.6	31–166
	14	31	199	541.9	
	21	102	145	42.2	
	28	124	155	25.0	
	35	67	66	–1.5	
	42	14	33	135.7	

* Reference ranges according to Garfia Veterinary Laboratory [40]. Δ%: Percentage difference of the SMC treatment relative to the Control treatment. Given *n* = 1 per week, data are presented for descriptive longitudinal monitoring.

A critical finding was the behavior of the liver enzymes Alanine aminotransferase (ALT) and Aspartate aminotransferase (AST). These enzymes are primary biomarkers of hepatocellular integrity and are released into the bloodstream upon liver damage [1, 46]. In this study, the SMC treatment exhibited a more stable enzymatic response during the transition to the Finisher phase. While the Control treatment showed an abrupt increase in liver enzyme activity by Day 35 (ALT: 260 UI/l; AST: 270 UI/l), the SMC treatment maintained lower levels (Δ = –57.7% for ALT and –55.6% for AST) during this window of high metabolic demand. This suggests that the symbiotic complex effectively buffered the hepatic effects of environmental stressors, including high temperature (29.6°C) and elevated water nitrate levels. Although treatment converged toward higher values by the end of the study (Day 42), the stability provided by the SMC during the growth peak points to enhanced metabolic resilience. This is consistent with Shanmugasundaram et al. [48] and Ogbuewu et al. [38], who emphasize that *Bacillus*-based probiotics provide homeostatic support and act as hepatoprotective agents in birds challenged by environmental or nutritional stress.

As stated by Bogatko [41], fluctuations in serum biochemistry are expected during probiotic inclusion as part of the metabolic adaptation to improved nutrient absorption. This is particularly relevant given that *Bacillus clausii* and *Bacillus subtilis* enhance the bioavailability of nutrients [33], supporting a robust metabolic status necessary to face the environmental challenges monitored in this study [52].

Regarding lipid profiles, the SMC treatment showed numerically higher cholesterol and triglyceride levels than the Control treatment (Table 4). While some literature suggests that probiotics typically exert a hypocholesterolemic effect [53], our findings indicate that the SMC maintained these parameters within the updated physiological reference intervals for modern Ross broilers, as recently established by Zálešáková et al. [42].

The observed levels do not represent a metabolic disorder but rather a state of homeostatic stability under rearing stress. Other studies also demonstrate the sensitivity of these indicators to dietary interventions [1, 2]. Although some components, such as β-glucans, are associated with lowering cholesterol and triglycerides [12, 54], environmental stressors can modulate this response. Furthermore, according to Bogatko [41], fluctuations in serum biochemistry are expected during probiotic supplementation as part of the metabolic adaptation to improved nutrient absorption. This is particularly relevant given that *Bacillus clausii* and *Bacillus subtilis* enhance the bioavailability of nutrients [33]. Therefore, the lipid profile in the SMC treatment likely reflects a robust metabolic status necessary to face the environmental challenges recorded in this study, rather than a lipid imbalance [35, 52].

## 4. Conclusions

The effectiveness of SMC was demonstrated through the synergy between its probiotic (*Bacillus* spp.) and prebiotic (β-glucan and β-mannans) components, which significantly improved bird viability by reducing early mortality. In response to the recorded environmental conditions, it is evident that the birds faced a multifactorial stressor scenario, including high ambient temperatures (29.6°C) and the presence of nitrates in the water. This combination of stressors, along with a natural infectious challenge, imposed a significant physiological toll, as reflected in the hematological and biochemical deviations observed in both treatments. Under these challenging conditions, the protective effect of the SMC acted as a metabolic and immunological buffer rather than a growth promoter. While the treatment did not result in superior weight gain compared to Control, it provided essential homeostatic support-stabilizing liver enzymes (ALT/AST), maintaining lymphocyte dynamics, and ensuring survival rates consistent with breed standards. Regarding animal health and welfare, the maximum potential for weight gain and feed intake is likely optimized when combined with rigorous farm management, particularly in terms of water quality and climate control. Future research should evaluate the SMC under strictly controlled environmental conditions to decouple its growth–promoting potential from its proven survival–enhancing capabilities.

## Data Availability

The data presented in this study are available from the corresponding author upon reasonable request.

## List of abbreviations

SMC, symbiotic microbial complex; COBIANIM, Bioethics Committee for Animal Research of the Venezuelan Institute for Scientific Research; IBW, initial body weight; FBW, final body weight; DFI, daily feed intake; BWG, body weight gain; FCR, feed conversion ratio; SD, standard deviation; ALT, alanine aminotransferase; AST, Aspartate aminotransferase; MOS, mannan-oligosaccharides.
